# *Omics* data in relative values are almost subcompositionally coherent

**DOI:** 10.3389/fmicb.2026.1809364

**Published:** 2026-07-23

**Authors:** Marina Martínez-Álvaro, Michael Greenacre, Agustín Blasco

**Affiliations:** 1 Instituto de Ciencia y Tecnología Animal, Universitat Politècnica de València, Valencia, Spain; 2 Department of Economics and Business, Universitat Pompeu Fabra, Barcelona, Spain

**Keywords:** compositional data, logratio transformation, *omics*, subcompositional coherence, supervised learning, unsupervised learning

## Abstract

**Introduction:**

*Omics* data are compositional and often expressed as relative abundances after total sum scaling normalization. An important statistical issue with compositional data is the lack of subcompositional coherence, meaning that relative abundances change when data are re-normalized after removing or adding features. While this problem is well documented for small compositions, it has not been investigated in large *Omics* datasets, which typically contain hundreds or thousands of features and where subcompositions are ubiquitous. Subcompositions arise, for example, when using different reference datasets, sequencing depths or when filtering low-abundant features from the database. In such cases, the most abundant features are preferentially retained, whereas variation between original or full compositions and subcompositions is mainly driven by less abundant features. The standard solution to this problem is the use of logratio transformations, but these complicate interpretations and require handling zeros, which are frequent in *Omics* data and whose imputation introduces spurious variability.

**Methods:**

Here, we evaluated subcompositional coherence in five representative *Omics* datasets: fecal 16S metagenomics, rumen metagenomics (taxonomic and functional levels), liver transcriptomics, and plasma metabolomics, considering both unsupervised and supervised learning contexts. We generated 100 random subcompositions comprising one-third of the original features under an abundance-weighted subcomposition scheme and compared their statistical outputs with those from the full composition.

**Results and discussion:**

Raw *Omics* data showed near-perfect coherence: relative abundances, pairwise correlations and sample distances all exhibited very high (scaled) concordances (≥0.98–0.99). Outputs from commonly used supervised models (linear regression, PLS, random forest, and linear mixed models with a Gaussian kernel) were also highly subcompositionally coherent. We conclude that large *Omics* datasets expressed as relative abundances are almost subcompositionally coherent when considering a weighted subcomposition scheme, thereby challenging one of the criticisms of using relative data in the *Omics* field over logratio transformations.

## 1 Introduction

The features of the various *omics* fields, such as microbiomics, metagenomics, transcriptomics, and semi-quantitative metabolomics, typically measured in counts, peak intensities, or any other non-negative measurements, are constrained to one another. This is due to many different steps of the wet-lab protocol such as matrix sampling, DNA extraction, PCR amplification, library preparation, each involving subsetting fixed volumes, and also to instrument's sequencing capacity or total ion capacity, each instrument delivering a restricted number of sequenced reads or ion counts. Each of these steps imposes its own constraint, so the nature of *omics* features is compositional, carries only relative information and the absolute scale is lost. The library size, which can be computed as the total number of reads after sequencing, counts or ion counts obtained, differs between samples, is not related to the absolute load in the original sample, but to other technical aspects such as extraction efficiency or instrument sensitivity. The removal of this systematic variability is attempted through different normalization methods. The simplest approach used is normalization by total sum scaling (TSS), also named as total ion current in metabolomic studies, where the features are usually expressed as relative abundances to the total sum of counts per sample. This procedure leads to compositions that sum to 1 or 100% for each sample and removing any explicit information about sequencing depth. Other normalization methods, such as trimmed-mean of M-values (TMM, Robinson and Oshlack (2010)), relative log-expression (RLE, Anders and Huber (2010), or cumulative sum scaling (CSS, Paulson et al. (2013), estimate sample-specific size factors, preserving some information about differences between samples in library size or total ions. These factors are derived from the observed original counts, and the normalized relative values remain compositional.

One important statistical issue with compositional datasets is that the relative values of the features change when a subset of features (a subcomposition) is selected and re-normalized; that is, they are subcompositionally incoherent. Subcompositions are widespread in the *omics* field: theoretically, the set of *omics* features detected in a given sample is always a subcomposition of a larger, underlying set of features present in the original system. In practice, subcompositions in the *omics* field arise whenever the same collection of DNA fragments is sequenced using different platforms or at different sequencing depths, as lower depth decreases the detection of rare features (Zaheer et al., 2018). Subcompositions also arise when sequenced reads are reprocessed against different reference databases, or when features of the datasets are filtered out due to low prevalence, low abundance, or unknown biological function, and the dataset is re-normalized. In all these practical cases, the most abundant features are preferentially retained, whereas variation between original or full compositions and subcompositions is mainly driven by less abundant features. Other desirable compositional behaviors that relative values must satisfy include scale invariance (multiplying all raw counts of a sample by a random integer number should not affect the posterior results) and perturbation invariance (distances between samples should not be affected when a subset of features has a systematic scaling) (Aitchison, 1992). If *omics* datasets in relative values do not achieve subcompositional coherence, scale invariance, and perturbation invariance, the reproducibility of the results can be seriously compromised.

Aitchison proposed the use of pairwise ratios between features as the appropriate data transformation for analyzing compositional data, preferably log-transforming the ratios to bring the ratios into real space and closer to normality, giving logratios (Aitchison, 1986). Pairwise logratios are subcompositionally coherent, as they remain invariant to the number of features in the dataset and to the sum of totals within a sample; and they are also scale and perturbation invariant. However, while logratios solve all these problems, their use has created additional problems. First, *omics* data have hundreds or thousands of features, so the number of pairwise logratios generated could become hard to handle, but more importantly, overwhelming to interpret. This can be bypassed using centered logratios (CLR), which preserves the geometry between samples, but they are not subcompositionally coherent. Alternatively, the additive logratio (ALR) transformation is subcompositionally coherent and can also approximately preserve distances when an appropriate reference feature is chosen, found to be naturally present in different *omics* datasets (Greenacre et al., 2021). Second, the log transformation requires handling zero values because zeros cannot be in the denominator of a ratio, and when they occur in the numerator, the resulting ratio of zero is also problematic for log-based transformations. This situation can be addressed using alternative approximations instead of logarithms, such as the arcsine (Zhang et al., 2024) or power transformations (Greenacre, 2024), although both are difficult to interpret. In fields where the occurrence of zeros is low, they can be replaced by small positive numbers, for example, some fraction of the detection limits in chemical applications. It is also common to automatically replace them by some more sophisticated imputation method – for an overview, see Lubbe et al. (2021). For the *omics* field, however, there are typically very large numbers of zeros, which can be as much as 80-90% of the whole data set, and then the strategy of zero replacement introduces spurious variability in the data (Kaul et al., 2017; te Beest et al., 2021).

Gloor et al. (2017) highlighted the compositional nature of *omics* data, warned about their subcompositional incoherence when expressed in relative values and encouraged the use of logratio transformations. However, the lack of subcompositional coherence in relative values of compositional data has usually been illustrated with small datasets, far from the realistic large *omics* datasets. For example, Egozcue and Pawlowsky-Glahn (2011) give an extreme example with three features to show how seriously the correlations are affected between the features when the third feature is dropped and the data are TSS-renormalized. Similar examples are given by Gloor et al. (2017) (supplement) and Greenacre (2018) to show the problem. These illustrations are certainly convincing, but they are not representative of the *omics* situation being considered here, namely datasets with hundreds or thousands of features. It would be valuable to assess how subcompositional incoherence behaves in high-dimensional contexts and whether this issue gets diluted and can be safely ignored, or needs to be explicitly addressed.

In a later study published in 2023, Gloor (2023) proposes five simple tests to evaluate whether *omics* datasets under different transformations express compositional behavior. Subcompositional incoherence was tested by checking whether correlations between common features were similar in the full dataset and in a subset of features. However, it was not clear whether small deviations from perfect agreement between feature correlations might affect the results of downstream supervised analyses, which is an aspect we aim to investigate. Subcompositional coherence can be tested by forming subcompositions of several *omics* datasets (microbiomics, metagenomics, transcriptomics, and metabolomics), and comparing them to the original composition (referred to as the full composition). The simplest form of comparison is to assess whether the set of values of an *omic* feature remains coherent with itself, named feature coherence. In an unsupervised learning context, we can compare the level of agreement in overall geometries between features, Pearson correlations between features (often used as counterexamples advocating against not using logratio transformed compositional data), or distances between samples. The case of supervised learning is different, where concern is focused on the results of the prediction of a response feature. For example, do the differential abundances remain coherent? Do the coefficients and their associated *p*-values change? Do the feature importances change in projection to latent structures regression (PLS) or in random forest? Is there coherence in the variance explained by an *omic* random effect in a linear mixed model with a Gaussian kernel? Or does subcompositional coherence also extend to prediction accuracy when the model is fitted and evaluated using a train–test split?

The objective of this work is to comprehensively evaluate subcompositional coherence in several *omics* datasets expressed in relative values after TSS normalization. We aim to elucidate whether *omics* datasets expressed in relative values can be considered sufficiently coherent for practical analytical purposes, and thus challenge one of the reasons why logratio transformations are required.

## 2 Materials and methods

### 2.1 *Omics* datasets

**Figure 1:**
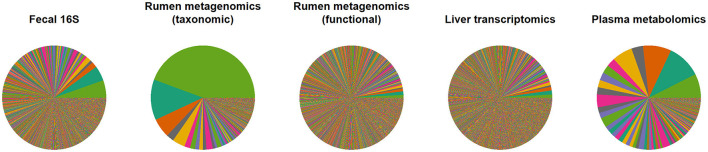
Description of the five *omic* databases. Portions represent the mean relative abundance of the *omic* features across the samples.

We used five different *omic* datasets: ruminal metagenomics in beef cattle resolved at taxonomic and functional levels, and liver transcriptomics and plasma metabolomics in rabbits. In all cases, all *omic* features with zero values in all the samples were eliminated before any analysis. Figure 1 provides an overview of the composition of the 5 datasets, which are described as follows:

*Soft fecal 16S metagenomic sequencing*: This is a database with the abundances of *m* = 19,205 Amplicon Sequence Variants (ASVs) obtained from sequencing the V3 and V4 regions of the bacterial 16S rRNA gene in feces samples from 199 rabbit does (Biada et al., 2025). Females belonged to two different commercial maternal rabbit lines and feces were collected immediately after first partum in two different seasons, 97 in summer and 102 winter, in a balance line-season experimental design. The litter size (number of kits alive) at the first partum was recorded. More details about the experiment and samples processing can be found at Biada et al. (2025). From all the values (199 × 19, 205 = 3, 259, 394), this database contains 92.2% zeros. In this database, supervised analysis was performed to find ASVs with differential abundance between seasons or with predictive ability for litter size.

*Rumen metagenomics in beef cattle*: This is a database of whole-metagenome sequenced rumen samples of 359 cows collected at SRUC's Beef and Sheep Research Center in Edinburgh, UK (Rooke et al., 2014; Roehe et al., 2016; Duthie et al., 2017). Sequences were processed with two bioinformatic pipelines to obtain the taxonomic (composition of microbial genera) and functional (composition of microbial genes) profiles. The two resultant databases were treated separately. The taxonomic composition was composed of abundances of *m* = 1,178 microbial genera resolved by the pipeline described in Mattock et al. (2023). From all the values (359 × 1, 178 = 422, 902), this database contains 0.25% zero values. Functional composition was composed by *m* = 6,750 microbial genes (after removing all uncharacterized counts, often labeled as No Hit), resolved by the pipelines described in Martínez-Álvaro et al. (2024). Of all the values (359 × 6, 750 = 2, 423, 250), this database contains 37.88% zeros. In both databases, supervised analysis was performed to find microbial genera or microbial gene abundances differentially abundant between dietary groups (253 and 106 animals were fed with either forage or concentrate-based diets) or explaining the response variable average daily gain (ADG), computed as a linear regression between weekly body weights and days during 4 months, when the animals are aged between 394 ± 32 and 505 ± 33 days. More details can be found in Martínez-Álvaro et al. (2024).

*Liver transcriptomics in rabbits*: This is a database with *m* = 25, 502 transcript abundances from the livers of 48 rabbits. The rabbits have two different genetic backgrounds: 24 were selected for high amount of intramuscular fat (IMF) and 24 for low IMF for 9 generations. Details of the sequencing are described in Valdés-Hernández et al. (2025). From all the values (48 × 25, 502 = 1, 224, 096), this database contains 36.09% of zeros. In this database, supervised analysis was performed to find transcripts with differential abundance between genders (24 males and 24 females) or to predict IMF based on transcript abundances. Details of the selection procedure and IMF content of the lines are described in Martínez-Álvaro et al. (2016) and Zubiri-Gaitán et al. (2023).

*Plasma metabolomics in rabbits*: This is a database of *m* = 997 metabolite abundances from the plasma of the same 48 rabbits with transcriptomic data, coming from untargeted analysis using Ultra-Performance Liquid Chromatography coupled to Tandem Mass Spectrometry (UPLC-MS/MS) already published in Zubiri-Gaitán et al. (2023). Abundance of metabolites were measured as unnormalized peak areas (i.e., total ion counts, integrated area-under-the-curve). From all the values (48 × 997 = 47, 856), this database contains 4.7% of zeros. In this database, supervised analysis was performed as mentioned for the liver transcriptomics.

### 2.2 Definition of relevant subcompositions

**Figure 2:**
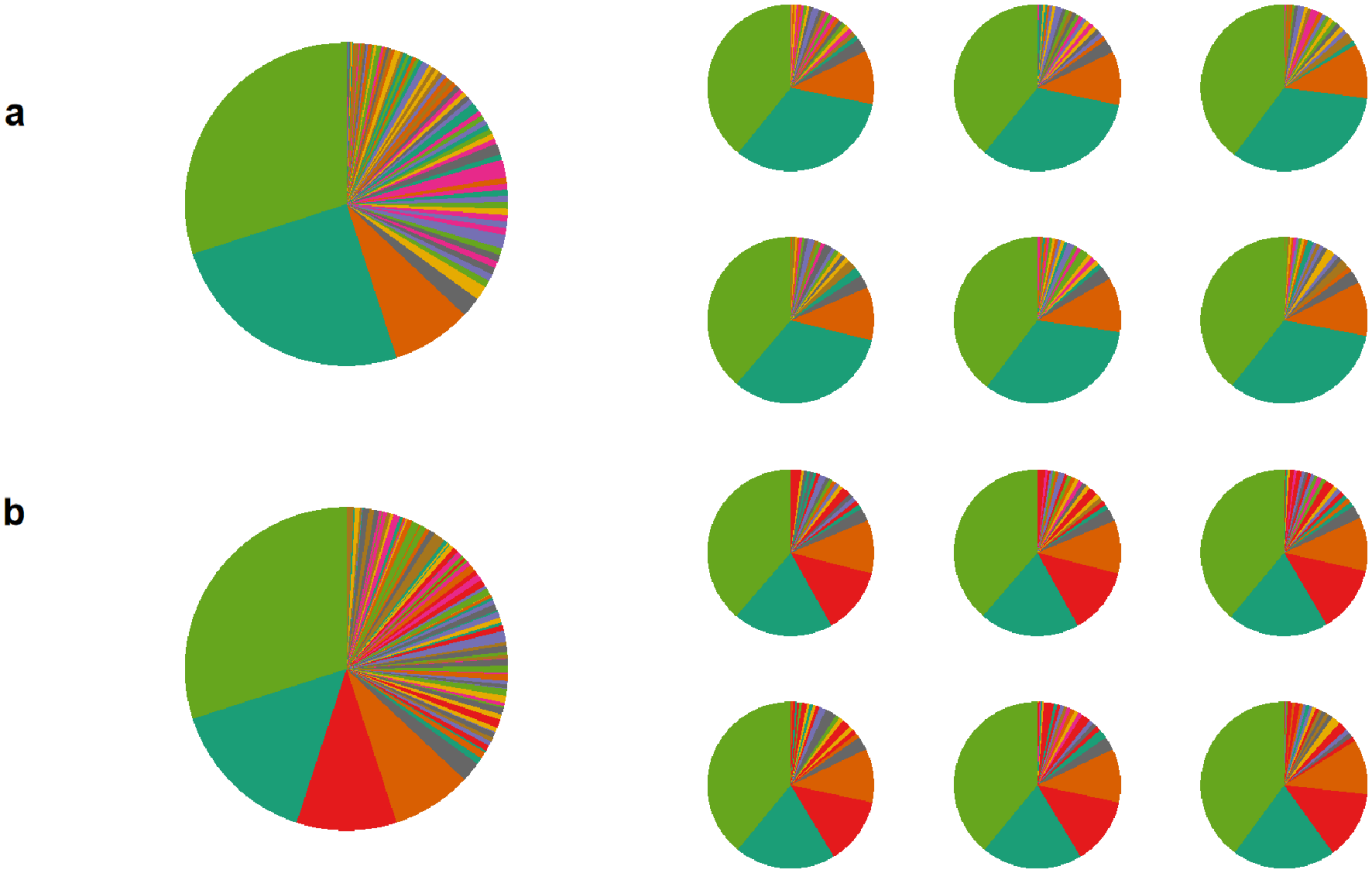
Graphical example of how subcompositions are simulated from the full composition. Large pies represent the full set of features or full composition; small pies represent the resulting subcompositions. In **(a)**, subcompositions are generated in an unsupervised context through weighted sampling, so abundant features are more likely to be included than rare ones. In **(b)**, subcompositions are generated in a feature-selection supervised context: the red segments correspond to features selected as important for the prediction in the full composition and are always included, while the remaining features are added through weighted sampling.

In all cases, we use as reference the dataset including all defined *omic* features expressed in TSS-normalized relative abundances, so that the total sum within each sample equals 1, from now on referred to as the full composition. To simulate realistic scenarios of partial data availability, we generate random subcompositions by selecting a subset of *omic* features and TSS-renormalizing the data (so each sample again sums to 1), as if these were the only available features (Figure 2). We generated 100 such random subcompositions per dataset to assess the variability of the results in different random feature selections. We fixed the size of each subcomposition to one-third of the full feature set (denoted as *m*_*s*_ = 1/3 *m*). This proportion represents a conservative scenario, as it is highly unlikely that any *omics* dataset will include so few parts, and larger subcompositions would likely yield even stronger coherence. The number of features selected in each *omics* dataset were 6,401 ASVs (out of 19,205), 2,250 microbial genes (of 6,750), 393 microbial genera (of 1,178), 8,500 transcripts (of 25,502), and 332 metabolites (of 997). We also include a subcompositional coherence test when using different subcompositional sizes (considering sizes 10%, 20%, 30%, 40%, 50%, 60%, 70%, 80%, or 90%) in Supplementary material as in Greenacre (2024). *Omics* datasets typically consist of hundreds or thousands of features, ranging from highly abundant to extremely rare. In follow-up studies or comparative experiments, it is common for abundant features to be included, but would probably differ in which rare parts are included. To reflect this, we weighted the feature selection probability by each feature's average relative abundance, making abundant features more likely to be selected than rare ones (Figure 2), and our results should be interpreted within this context. This sampling strategy is most relevant for datasets with highly uneven abundance distributions (e.g., microbial genera, 16S amplicon sequencing, and metabolomics), and less so for datasets with more homogeneous feature abundances (e.g., transcriptomics and functional metagenomics).

For the predictive task in the supervised learning context, we needed to slightly modify the way we created the subcompositions. Suppose that $m_{s}^{*}$ are the *omic* features selected in a predictive model as main contributors. Thus, *m*_*s*_ - $m_{s}^{*}$ features are randomly added to the $m_{s}^{*}$ fixed set of features by weighted sampling, the subcomposition is renormalized and then the model re-estimated using the subcompositional values of the $m_{s}^{*}$ features. Importantly, the number of samples remains constant in all cases.

### 2.3 Measures of agreement

To assess the level of coherence between the common set of *omic* features in both datasets, the full composition and each subcomposition, we compare their outputs using three measures of agreement: concordance, scaled concordance, and Pearson correlation. Supplementary Figure S1 illustrates these three measures using an example of an *omic* feature normalized in a full composition and its values normalized in three different subcompositions.

The concordance coefficient (Lin, 1989) measures exact numerical agreement between two sets of observations. Geometrically, it reflects how closely the points in a scatterplot align with the 45-degree line (a line through the origin with slope 1). Thus, the concordance is a stricter measure than the standard Pearson correlation coefficient, which captures closeness to any linear relationship, regardless of slope or intercept. The concordance coefficient between two vectors *x* and *y* (both *n* x 1) is defined as:


$$\begin{array}{l} C=\frac{2r_{xy},s_{x}s_{y}}{s_{x}^{2}+s_{y}^{2}+{\left(\bar{x}-ȳ\right)}^{2}} \end{array}$$ (1)

where *r*_*xy*_ is the correlation between *x* and *y*, *s*_*x*_ and *s*_*y*_ are the standard deviations of *x* and *y*, and $\bar{x}$, ȳ the respective means. The concordance has a maximum of 1 and will be equal to 1 only if the two vectors are numerically identical. Scaled concordance applies a scaling factor, and then computes concordance on the adjusted values. The scaling factor is obtained from the slope of the regression through the origin of *y* on *x*, which is equal to $b=\underset{i}{\sum }x_{i}y_{i}/\underset{i}{\sum }x_{i}^{2}$. Hence, scaled concordance is the concordance function (1) applied to *bx* and *y*.

### 2.4 Subcompositional coherence under different contexts

As discussed in the introduction, the meaning of subcompositional coherence varies depending on the specific research objective and differs between unsupervised and supervised learning contexts. In this section, we outline several ways to assess coherence within *omics* research. Two functions programmed in R, named unsuper_subco() and super_subco(), are designed to test subcompositional coherence and is publicly available on GitHub (see Data Availability section).

### 2.5 Feature coherence

Since *omic* values in subcompositions are obtained by TSS-normalizing subsets of data with respect to different totals, they inevitably increase compared to their values in the full composition. To assess how much they change, independently of the learning context, we first quantify the concordance of the values of each *omic* feature when normalized in the full composition and in a subcomposition. Even if concordance is not very high, coherence could still be exact if the subcompositional *omic* values were just a constant scale factor increase of the full compositional values. This makes scaled concordance especially relevant, as they are robust to global rescaling. To explore this, we computed concordance and scaled concordance for each *omic* feature by comparing its values in the full composition and in 100 randomly selected subcompositions. Each *omic* feature comparison yielded a coefficient, and the results were averaged across the 100 subcompositions, obtaining average agreement measures for each *omic* feature. We also tested the variation in feature scaled coherence when considering subcompositions with different subcompositional sizes (10%, 20%, 30%, 40%, 50%, 60%, 70%, 80%, or 90% of the total number of features in the full composition) and when increasing the number of subcompositions sampled from 100 to 1,000.

### 2.6 Coherence in an unsupervised learning context

In a matrix of *m* features for *n* samples, unsupervised analyses often rely on the relationships between features, such as pairwise distances, correlations, covariances, or on the geometry of the features. The instability in the relationships between *omic* features in subcompositions is the main concern in the analysis of compositional data. Here we tested the coherence of the complete geometry of the composition (using Procrustes correlation), as well as the coherence of pairwise Pearson correlations, covariances and Euclidean distances between *omic* features. We also tested the similarity between full composition and subcompositions of pairwise Euclidean distances between samples and within-sample Shannon and Simpson indices.

Procrustes correlation was used to assess the geometric coherence between features in the subcomposition and the full composition. This method aligns two multidimensional configurations, defined by principal coordinates of the *omic* features in the subcomposition and in the full composition, by translation, scaling and rotation to match each other, using singular value decomposition (see Greenacre (2018) and Greenacre (2024) for mathematical details). The number of dimensions will be *n*−1 in both configurations as *n* < *m*_*s*_. The resulting Procrustes correlation is the correlation between the two sets of coordinates (where one set has been fitted to the other by Procrustes analysis). This correlation is computed on the two sets that have been vectorized into two vectors. Furthermore, in this case, the Procrustes correlation is identical to the concordance between the two vectors.

Euclidean distances, covariances, and Pearson correlations between pairwise *omic* features were computed to evaluate the coherence on the pairwise relationships between *omic* features in each subcomposition relative to the full composition. Each subcomposition yields a vector of *m*_*s*_(*m*_*s*_ − 1)/2 distances or correlations, compared with their counterparts in the full composition. In the case of covariances, variances were also included in the comparison, increasing the vector length to *m*_*s*_(*m*_*s*_ − 1)/2 + *m*_*s*_. As the coherence of pairwise correlations between *omic* features is especially relevant, we quantified their agreement using the coefficient of concordance, which provides a strict measure of coherence. Because renormalizing a subcomposition generally increases the magnitude of the values, distances and covariances among *omic* features are also expected to increase. Thus, in this case we choose the scaled concordance coefficients to assess their level of agreement. To further contextualize the variability due to subcompositions, we generated a bootstrap sample from the full composition and compared the resulting between-*omics* pairwise Pearson correlations to those from the original full composition, providing a reference for expected sampling variation.

If Procrustes correlations and relationships between features are coherent, then similarity in the geometry of the samples should be a direct consequence. We computed the Euclidean distances between samples, and each subcomposition yielded a vector of *n*(*n* − 1)/2 distances, compared to their counterparts in the full composition; and we also computed two measures of within-sample variation (Shannon and Simpson indices), each one yielding a vector of size *n*. For the three metrics, we chose the scaled concordance coefficient to assess their level of agreement

### 2.7 Coherence in a supervised learning context

In supervised learning, we considered two tasks: differential abundance analysis and prediction. Subcompositional coherence now signifies the similarity of the results pertaining to significant subcompositional features with the corresponding results of the same features in the full composition. Subcompositional coherence in differential abundance was evaluated by determining whether the same features with same effect sizes were identified between experimental conditions (diet in cows and gender or seasons in rabbits) in the full composition and across 100 subcompositions. Differential abundance was assessed using Wilcoxon rank-sum and t-tests with a *p*-value < 0.05, and the magnitude of differences was evaluated through effect size estimates.

In the predictive task, the subcompositional coherence issue concerns whether the contribution of each *omic* feature to the model and the model predictive performance remains the same when predictors come from a full composition of *m* parts or from a subcomposition of size *m*_*s*_. We used three parametric models, linear regression, Projection to latent structures regression (PLS), and linear mixed model with a Gaussian kernel (commonly used by animal breeders), and a non-parametric one, random forest. All five aimed to predict a continuous variable: ADG (kg/day) for cows and IMF (g/100g of meat) or litter size (number of kits born alive) for rabbits datasets.

The procedure used to assess subcompositional coherence was the same for linear regression, PLS, and random forest, since these three algorithms are typically applied after a variable selection step. The linear mixed model with a Gaussian kernel follows a different pipeline, as variable selection is not commonly used in this approach; its procedure is explained later. First, the full compositional dataset was split, as usual, into a train set (3/4) and a test set (1/4), and this partition was kept constant across all algorithms. Second, each model was fitted and optimized within the train dataset of the full composition, including *omic* feature selection and refitting using only the selected features. Third, 100 subcompositions were generated, each containing the relevant subset of features (see Figure 2), but otherwise complemented by a different remaining set of *omic* features, and then all renormalized to their respective totals. Fourth, the final model was fitted using data from each subcomposition. The models were fitted with the same features in both the full composition and the subcomposition, as we aimed to test the coherence of subcomposition normalization, without confounding it with the effect of introducing additional sources of noise into the model. Coherence was assessed by comparing models fitted to the full composition with those fitted to the 100 subcompositions, specifically in terms of the contribution of each *omic* feature and predictive performance in the train and test sets. For computational efficiency, the transcriptomic dataset was prefiltered to retain features with absolute Pearson correlation coefficients ≥|0.40| with IMF, resulting in 1,484 transcripts. We also filtered out ASVs that had a zero value in more than 90% of the animals, keeping 3,173 ASVs for the predictive exercise. Specifications for each algorithm are as follows:

*Linear regression*: A linear regression model was fitted in the train set. Features were added to the model as predictors sequentially according to the criterion that minimized the Bayesian Information Criterion (BIC). The optimal number of *omic* features to include was determined through 10 repetitions of 5-fold cross-validation within the train set. Next, we re-fitted the same linear model (using the same selected features) on the train set of a subcomposition. From each linear model, we retained the standardized regression coefficients, their 95% confidence intervals, *p*-values, and the root mean squared error (RMSE) in both the train and test sets as reference measures for comparison. Since regression coefficients depend on the scale of the predictors, we standardized all predictors to have unit variance to avoid scale-related biases in comparing these coefficients, as model performance is independent of the scale of the predictors. To compare variation due to subcompositions with the variation arising from multiple random train-test sample splits of the dataset, we re-fitted the same linear model using 100 different train sets and evaluated it on the corresponding 100 test sets.

*PLS*: A PLS model was fitted to the train set using the R package *mdatools* (Kucheryavskiy, 2020). The PLS model was fitted on the chi-squared-transformed values of *omic* features. The optimal number of latent components was always determined by 10 repetitions of 5-fold cross-validation within the train, minimizing the RMSE in the validation folds. Once the model was fitted, *omic* features were selected according to two criteria: a Variable Importance for Projection (VIP) score > 1 and jack-knife confidence intervals of the regression coefficients not including zero (Zubiri-Gaitán et al., 2023; Martínez-Álvaro et al., 2024). A new PLS model was then refitted using only the selected *omic* features and the same procedure for the number of latent components. From this model, we retained the feature's PLS regression coefficients, VIP values and rankings, and the model's predictive performance (RMSE) in the corresponding train and test sets. Finally, 100 PLS models with the selected features as predictors were fitted using each of the 100 train sets derived from 100 different subcompositions and we kept the PLS regression coefficients, VIP values and rankings, and RMSEs in the train and test sets.

*Random forest*: First, a random forest was fitted to the train set of the full composition using the randomForest R package (Liaw and Wiener, 2002). We used default values of hyperparameters *ntree* (500), *mtry* (number of predictors/3) and *nodesize* (5). We selected a subset of *omic* features following the Boruta algorithm (Kursa and Rudnicki, 2010) as recommended by O'Connell et al. (2025), which compares the importance of each feature against a randomized feature and retains only those that consistently outperform their randomized versions across iterations. A new random forest model was then fitted using only the selected *omic* features. From this model, we kept *omic* feature importances estimated based on the % of increase of mean square error when the feature values permute, importance-based rankings and RMSE in the train and test sets for comparisons with the subcompositions. Finally, 100 random forest models were fitted using each of the 100 train sets derived from the 100 different subcompositions and the selected subset of features and we kept the same information as before for comparison.

*Linear mixed model with a Gaussian kernel*: In genomics and quantitative genetics, the proportion of phenotypic variance explained for a target trait by the *omics* is commonly assessed using a linear mixed model in which the *omic* component is treated as a random effect with one level per sample. This effect is assumed to follow a Gaussian distribution with mean 0 and covariance structure defined by a kernel matrix, the cross-product between the *omic* profiles of the samples (Ross et al., 2013)). We also evaluated subcompositional coherence within this framework, but the analysis was only feasible for the 3 microbiome datasets, as the liver transcriptomics and plasma metabolomic datasets (48 individuals) were too small to reach convergence. Each subcomposition was generated as in the unsupervised framework (Figure 2a) and renormalized. For comparison, the same selected features were extracted from the full composition using their full-composition normalization. The ADG or litter size values for the test set were masked (using the same split as in the other algorithms). A linear mixed model including the mean and the *omic* random effect was then fitted to each subcomposition and to its corresponding reduced full composition, using in each case a different kernel computed from either the subcomposition or the full composition after chi-square normalization. This process was repeated for 100 subcompositions. Models were fitted with Bayesian inference (Blasco, 2017) using the BGLR package (Pérez and de los Campos, 2014), with MCMC settings of *niter* = 100,000, *burnin* = 20,000 and *thin* = 50. The fixed effects (mean) were assigned flat priors (i.e., Gaussian prior with a null mean and a very large variance). The random variances were assigned default priors; i.e., scaled-inverse chi square distributions with the prior degrees of freedom (df0) equal to 5 and the prior scale parameter S0=var(Y)*(df0+number of traits+1)*R2, var (Y) being the phenotypic variance of the trait (ADG) and R2 being defined as the proportion of variance that one expects, a priori, to be explained by the regression with 0.5 as default value (Pérez and de los Campos, 2014). We retained the medians and the highest posterior density intervals containing the 95% probability of the marginal posterior distributions for the variance components (residual and *omic* variance) and the estimated *omic* values for each sample. We also computed the RMSE in both the training and test sets. The results of each subcomposition were compared with those obtained from its corresponding reduced full composition.

## 3 Results

### 3.1 Feature coherence

**Figure 3:**
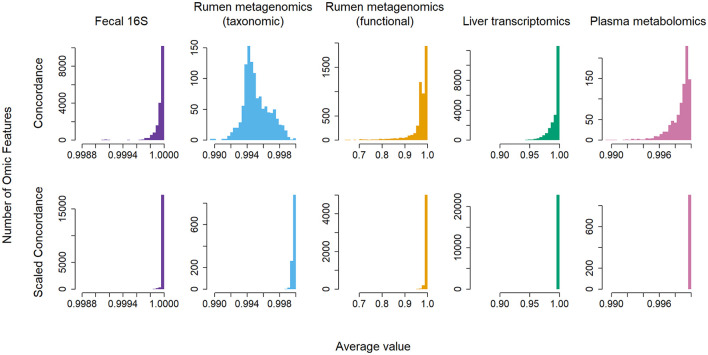
Coherence of each *omic* feature measured as the concordance or scaled concordance between its values when normalized in the full composition and its values when normalized within 100 randomly composed subcompositions.

Figure 3 displays histograms of feature coherence across five *omics* datasets. Overall, results indicate a very high degree of feature coherence. Strict concordance was nearly perfect for fecal ASVs, plasma metabolites and microbial genera. Concordance coefficients of the features had medians of 0.999, 0.999 and 0.995, respectively, and ranged from 0.989 to 0.999. In microbial gene abundances and transcriptomic features, concordances also showed very high medians, 0.979 and 0.994, but wider ranges and very asymmetrical concordance distributions. In the ASVs, microbial genera and plasma metabolite datasets, where certain components dominate the composition (e.g., *Prevotella* in rumen accounting for 44.3% of the total abundance; metabolite 799 in rabbits plasma representing 7.38%; and ASV1 in rabbit feces representing 5.39%, see Figure 1), the near-perfect concordance across *omic* features reflects that the features omitted in the subcompositions contribute very little to the total count, and the effect of renormalization is negligible. The few lower concordance values observed for some microbial gene abundances and transcriptomic features occurs because the features on these datasets have more uniform abundances (see Figure 1), causing some feature values to be substantially inflated when renormalizing within smaller subcomposition totals. In any case, these inflations were scalable. Scaled concordance was excellent across all datasets (all medians ≈ 0.999 and minimum scaled concordance was 0.958).

Supplementary Figure S2 shows how the coherence of *omic* features varies as the size of the subcomposition increases from 10% to 90% of the total number of features in the full composition. The average scaled concordance of *omic* features remains above 0.99 across all datasets, even under the most critical scenario (subcompositions of 10% of the total features). We focused on subcompositions comprising one third (33%) of the total *omic* features, as this represents a realistic yet critical scenario in practice.

Supplementary Figure S3 illustrates how *omic* feature scaled coherence varies when considering a larger number of subcompositions (up to 1,000). The main consequence of increasing the number of subcompositions tested is a gradual rise in the number of *omic* features included. Because of the weighted sampling, most but not all features were selected in the 100 subcompositions: 18,210 out of 19,205 ASVs (94.8%), 5,246 out of 6,750 (77.7%) microbial genes, 1,158 out of 1,178 (98.3%) microbial genera, 18,187 out of 25,502 (71.32%) transcripts and 904 out of 997 (90.67%) metabolites. The proportion of features selected increased gradually when moving from 100 to 1,000 subcompositions: 94.8% to 100% for ASVs, 98.3% vs. 99.9% for microbial genera, 77.7% vs. 87.0% for microbial genes, 71.3% vs. 89.4%) for transcripts and 90.7% vs. 99.6% for metabolites. Despite this increase in feature coverage, the average scaled feature coherence remained unchanged as the number of subcompositions increased from 100 to 1,000. Therefore, we considered 100 subcompositions sufficient for assessing coherence in the subsequent analyses.

### 3.2 Coherence in unsupervised learning

**Figure 4:**
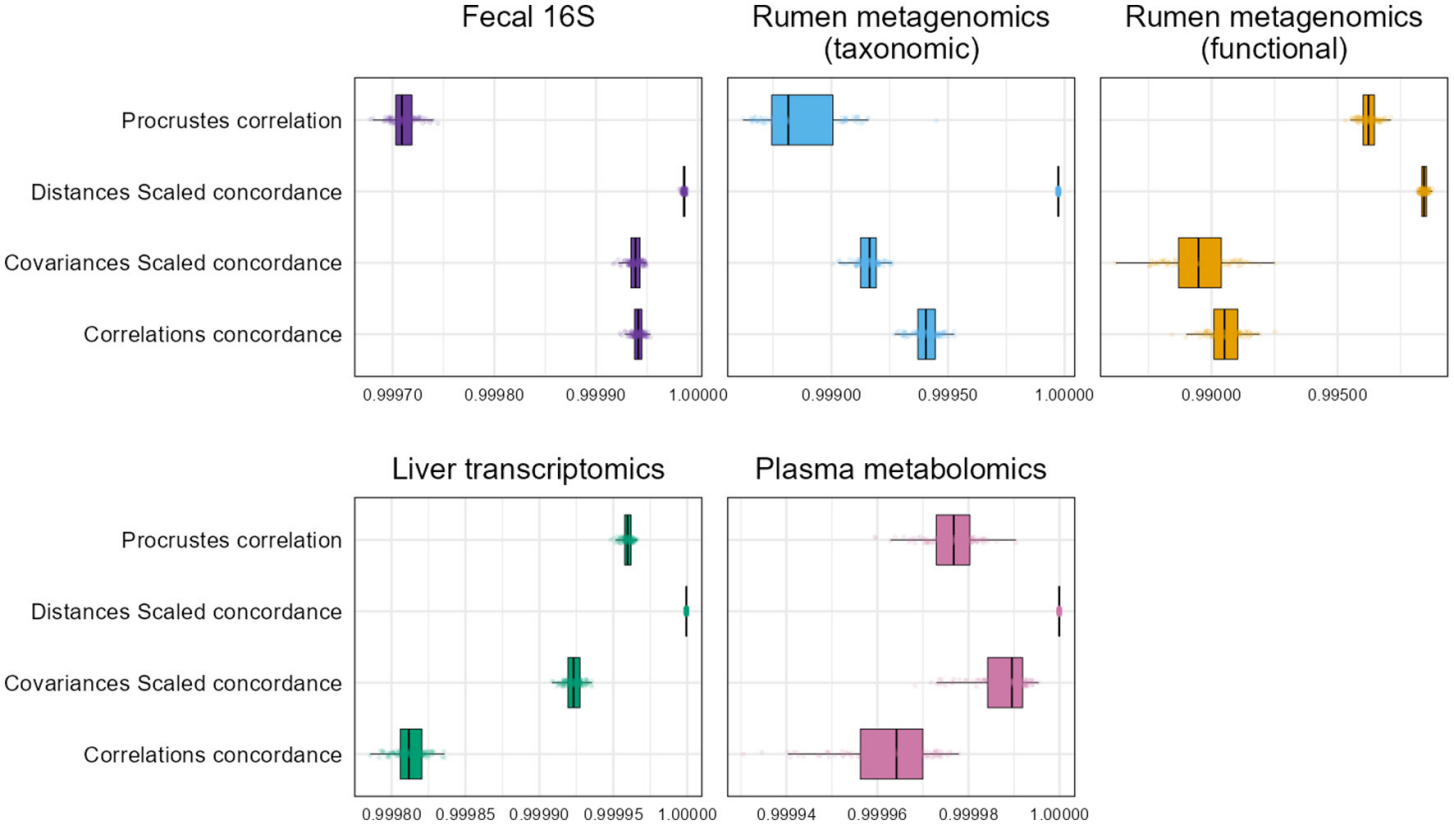
Subcompositional coherence of relationships between *omic* features in an unsupervised learning context. Each boxplot contains 100 values, each calculated from the agreement of the full composition and a distinct subcomposition. Procrustes correlation assesses coherence in the overall multivariate geometry while concordances evaluate the agreement on vectors composed by pairwise distances, covariances and correlations between *omic* features.

Figure 4 shows the subcompositional coherence of relationships between *omic* features, assessed across the 100 replicate subcompositions.

Results indicate near perfect coherence across the five datasets. Pairwise correlations between features were numerically concordant with nearly all values exceeding 0.989. Scaled concordances between pairwise distances and covariances in the subcompositions and the full composition were also consistently greater than 0.998 and 0.987, respectively. Procrustes correlations, which assess agreement in the overall multivariate configuration rather than pairwise relationships were greater than 0.995.

**Figure 5:**
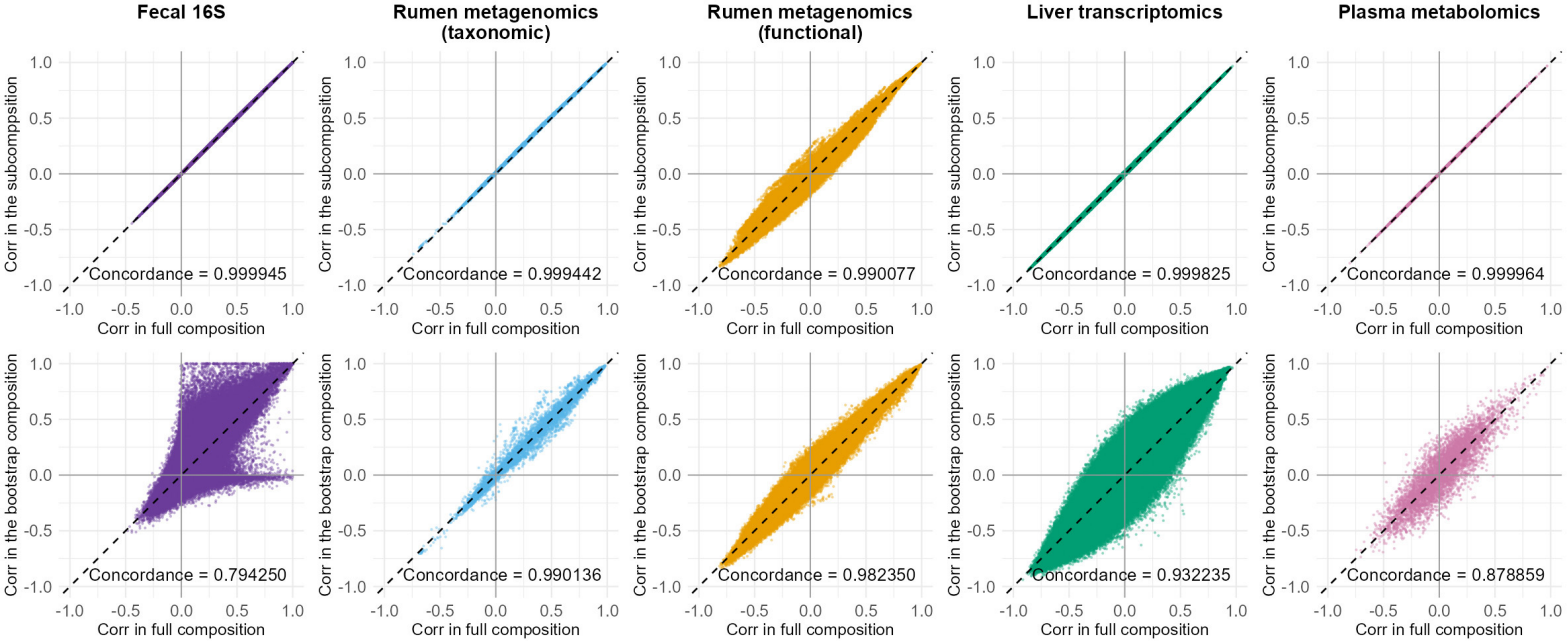
Scatterplots showing between features Pearson correlations variability in the full composition in comparison to their counterparts in one subcomposition or in a bootstrap sample.

Although the agreement between correlation coefficients is high in all cases, it is useful to examine this agreement graphically, to avoid spurious inflation due to the large number of pairwise correlations. The first row of panels from Figure 5 shows the scatterplots of all pairwise correlations in the first subcomposition tested and their counterparts in the full composition. Examination of these scatterplots shows a generally uniform level of agreement across datasets, with microbial genes showing slightly lower concordance. The variability of pairwise Pearson correlations between features due to subcompositional effects can be compared with the natural sampling variability of such correlations, which can be assessed from a bootstrap sample of the full composition. The second row of Figure 5 depicts the comparison with the bootstrap sample for the five datasets analyzed. In all cases, the variability of the correlations was greater in the bootstrap sample than in the subcomposition. The concordance between feature-wise correlations in the full composition and those in the subcomposition was consistently higher than the concordance between the full composition and its bootstrap sample. The values (subcomposition vs. bootstrap) were 0.999 vs. 0.990 for microbial genera; 0.990 vs. 0.982 for microbial genes; 0.999 vs. 0.879 for metabolites; and 0.999 vs. 0.932 for transcripts. Moreover, the proportion of correlations that changed sign in comparison to the full composition was always lower in the subcomposition than in the bootstrap sample. Very few correlations in the subcomposition (0.15%, 0.79%, 3.89%, 0.61%, and 0.24% for ASVs, microbial genera, genes, transcripts, and metabolites, respectively) lay in the upper-left or lower-right quadrants, indicating that sign reversals are rare and correspond to correlations close to zero. In contrast, the fractions of sign changes in the bootstrap samples increased by 14.07%, 2.02%, 6.36%, 11.07%, and 15.93% for the same datasets.

Given the high coherence in Procrustes and between *omic* features, coherence between samples is expected. Supplementary Figure S4 shows subcompostional coherence in the variation within a sample (Simpson and Shannon indices), and between samples (Euclidean distances). Samples on microbial genes dataset showed the largest within sample diversity among the five datasets (adjusted Shannon index was 0.842 on average), followed by transcriptomics (0.787), whilst ASVs, metabolites, and microbial genera were less diverse (0.684, 0.615 and 0.483), as anticipated in Figure 1. Simpson and Shannon indices were perfectly coherent with scaled concordances of 1 across the 4 datasets. Euclidean distances between the samples showed scaled concordances with medians greater than 0.998, and minimum values overall exceeded 0.99 (Supplementary Figure S4).

### 3.3 Coherence in supervised learning

**Figure 6:**
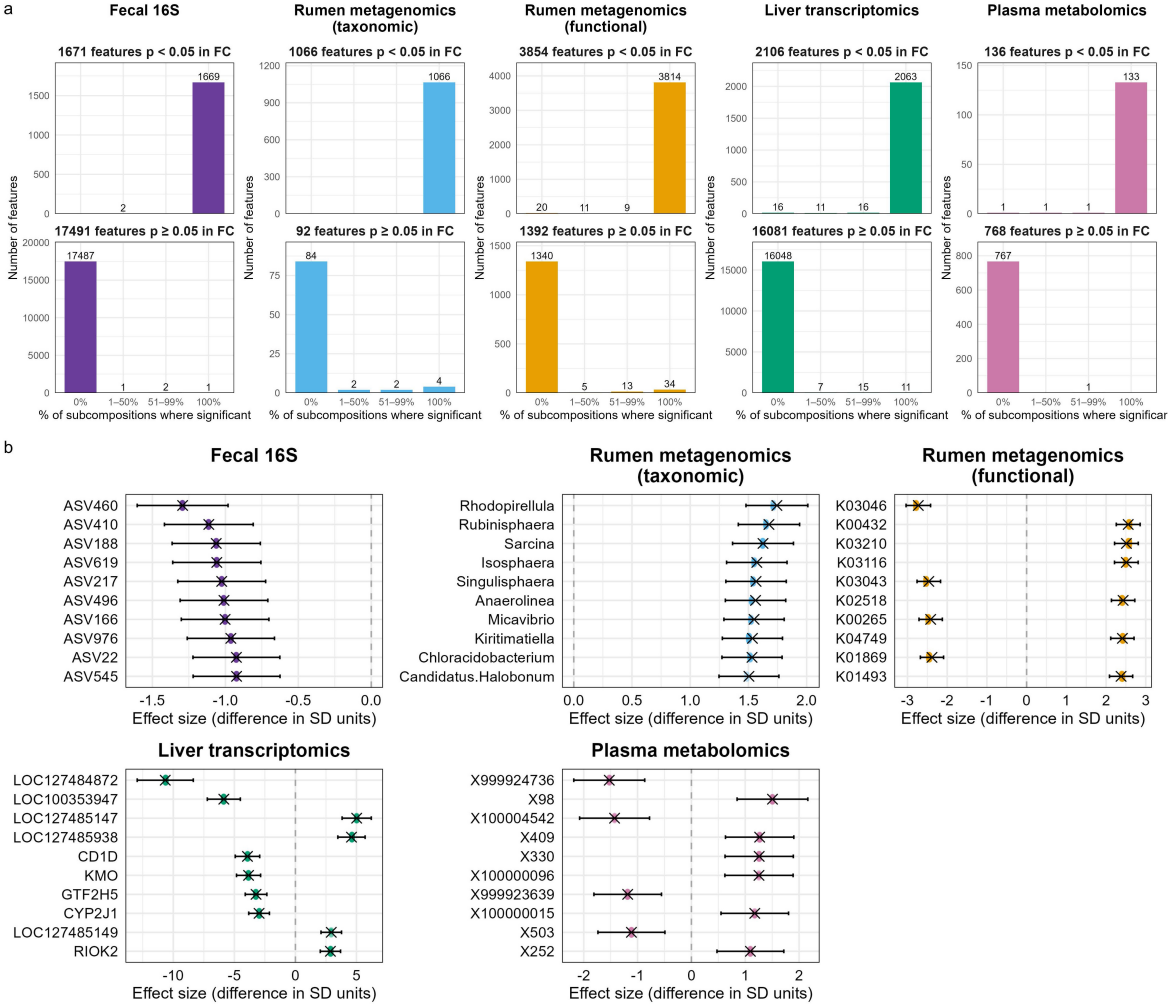
Subcompositional coherence in differential abundance analyses between seasons (ASVs), between diets (rumen microbial genera and microbial genes) or between genders (liver transcriptomics and plasma metabolomics). **(a)** Coherence on detection of differentially abundant features. Features were classified as significant or non-significant using a Wilcoxon rank-sum test with a significance threshold of *p* − *value* < 0.05. The upper and lower panels show the coherence of features identified as significant or non-significant in the full composition (FC), respectively. Bars indicate the percentage of subcompositions in which these features were identified as significant in the corresponding subcompositions. Features not selected in any subcomposition are omitted. **(b)** Coherence in effect sizes. Black crosses represent the estimated effect sizes of the top 10 *omic* features, together with their 95% confidence intervals. Colored points show the effect sizes when the feature is re-normalized in any of the 100 subcompositions.

The differential abundance analysis was subcompositionally coherent across all datasets. In the full composition, the Wilcoxon test detected 1,671 ASVs as differentially abundant between seasons, 1,066 genera and 3,854 microbial genes as differentially abundant between diets (the diet is known to have a large effect on the ruminal microbiome (Saez-Torillo et al., 2026)), and 2,106 transcripts and 136 metabolites as differentially abundant between genders (*p*-value < 0.05). In contrast, 17,491 ASVs, 92 genera, 1,392 genes, 16,081 transcripts, and 768 metabolites were not significant, while the remaining were not tested because they were not included in any subcomposition. In most cases, features identified in the full composition showed the same differential abundance result when they were re-normalized within a subcomposition (Figure 6a), and when discrepancies occurred, the corresponding *p*-values were very close to the significance threshold. A similar pattern was observed when differential abundance was assessed using a t-test (Supplementary Figure S5). Regardless of statistical significance, subcompositional effect sizes always fell within the 95% confidence interval of the effect size estimated from the full composition. As an illustration, the effect sizes obtained in the full composition and across subcompositions for the ten features with the largest effects in each dataset are shown in Figure 6b.

**Figure 7:**
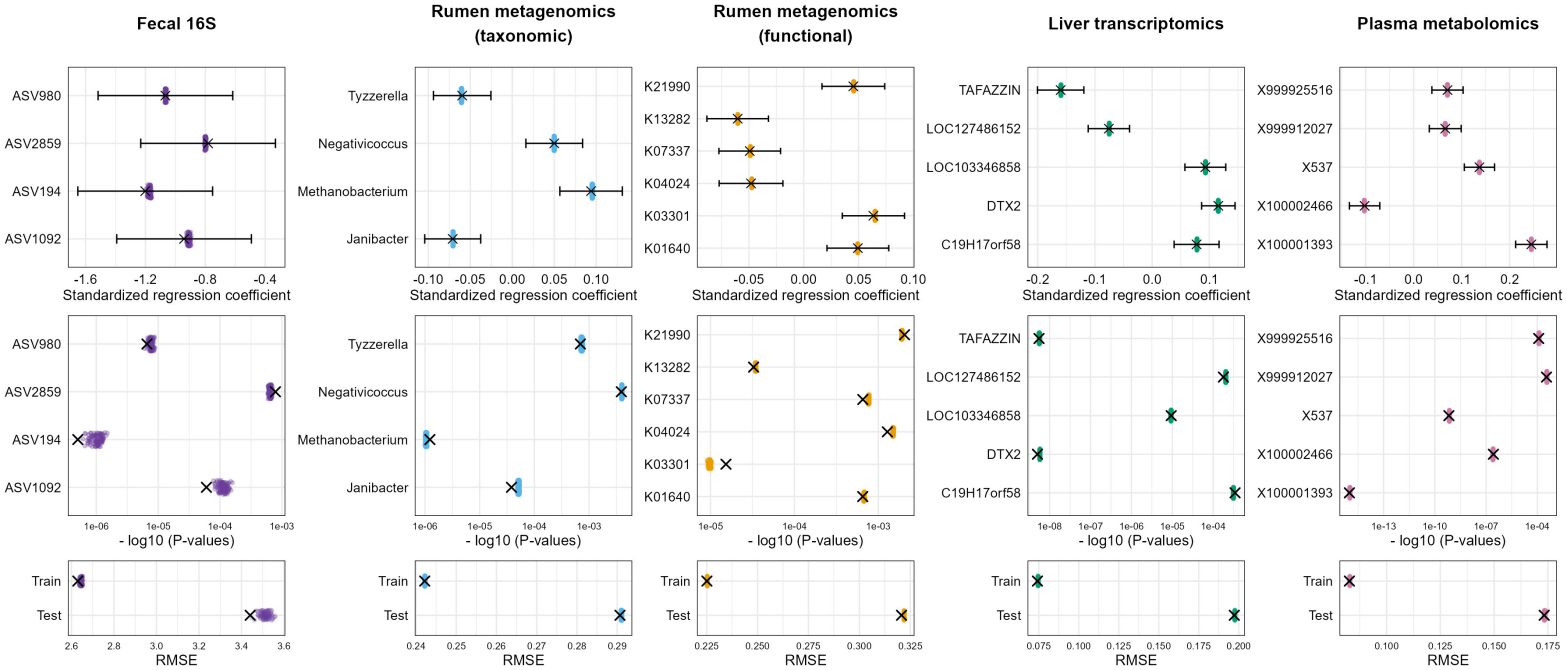
Subcompositional coherence analysis in five supervised linear regression models aimed at predicting litter size using ASVs, average daily gain using ruminal metagenomics (microbial genera or microbial genes) or intramuscular fat using liver transcriptomics or plasma metabolomics. Black crosses represent the estimated effect sizes of the selected *omic* features, expressed as standardized regression coefficients, together with their 95% confidence intervals, corresponding *p*-values, and the model root mean squared error (RMSE) for the training and test datasets, for a single data sample partition, when fitted using the full composition. Colored points show the same parameters obtained when the model is fitted using 100 different subcompositions.

**Figure 8:**
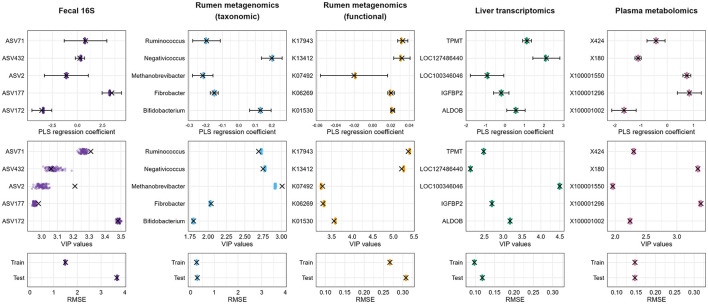
Subcompositional coherence analysis in five supervised projection to latent structures regression models aiming at predicting litter size using ASVs, average daily gain using ruminal metagenomics (microbial genera or microbial genes) or intramuscular fat using liver transcriptomics or plasma metabolomics. Black crosses represent regression coefficients of the five most important features in the prediction with their jack-knife intervals, their VIP (variable importance in the projection), and the model RMSE (root mean squared error) in the train and test dataset, for a single data sample partition, when the model is fitted with the full composition. In color, the same parameters obtained when the model is fitted with 100 different subcompositions.

**Figure 9:**
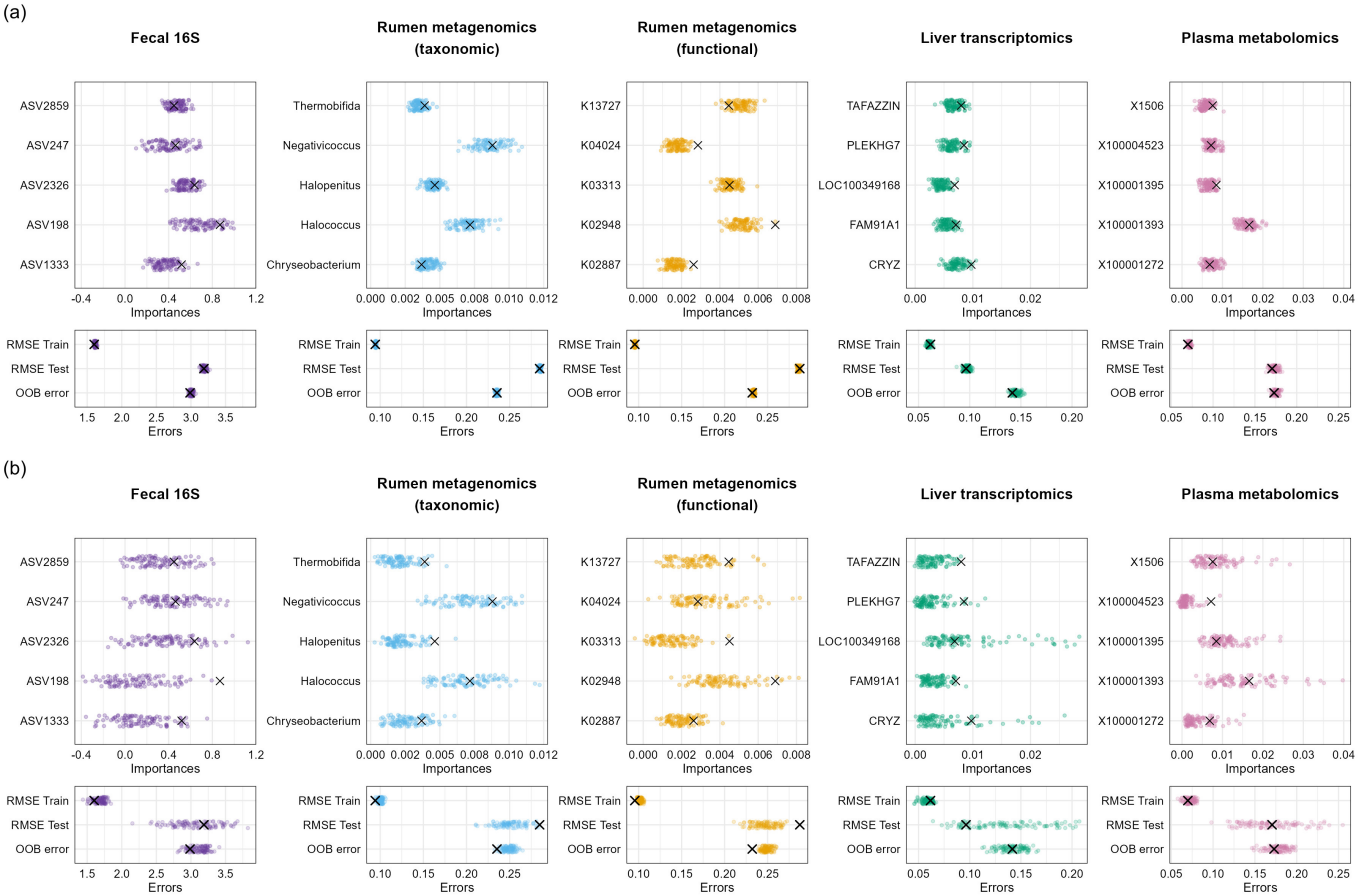
Coherence analysis in five supervised random forest regression models aiming at predicting litter size using ASVs, average daily gain using ruminal metagenomics (microbial genera or microbial genes) or intramuscular fat using liver transcriptomics or plasma metabolomics. Black crosses represent variable importances of five most important features in the prediction, and the model root mean squared error (RMSE) in the train, out of bag error, and RMSE in a test dataset, for the reference data partition used for each dataset along the whole manuscript, when the model is fitted with the full composition. The same parameters are obtained when the model is fitted with **(a)** 100 different subcompositions or **(b)** 100 train and test data splits.

Coherence in a predictive task was tested by comparing the fits of different parametric and non-parametric supervised algorithms when using the full composition or different subcompositions: linear regression (Figure 7), PLS (Figure 8), random forest (Figure 9) and Gaussian kernel linear mixed model (Supplementary Figure S6). Results show that for all datasets and for all algorithms tested, model parameters, features contribution to the model and model predictability on the train set and on the test set are stable with respect to using random subcompositions, leading to the same conclusions fitting the full composition or a subcomposition.

Linear regression models were fitted including a reduced set of *omic* features (4 ASVs, 4 microbial genera, 6 microbial genes, 5 transcripts and 5 metabolites) selected by stepwise regression on the full composition (Figure 7). Standarized regression coefficients ranged between –1.66 and 0.83 with *p*-values ≤ 0.004. Standardized regression coefficients and their *p*-values were coherent across the 100 subcompositions for all predictors, with only minor discrepancies observed such as for microbial gene K03301, which had a *p*-value of 1.53 × 10^−5^ in the full composition but slightly lower value in the subcompositions (range: 9.05 × 10^−6^ to 1.03 × 10^−5^; median: 9.79 × 10^−6^), while still being highly significant. Prediction performance (RMSE) in both train and test sets was nearly identical between the full composition and the subcompositions, and remained highly stable across the 100 subcompositions.

Subcompositional coherence in a supervised context was evaluated using the same train-test data split per dataset across all algorithms, ensuring that the variability introduced by subcompositional changes could be assessed independently from the variability arising from different data partitions. However, the variation observed across subcompositions can be compared with the natural variation in model parameters that occurs when different random train-test splits are used, as commonly recommended in machine-learning workflows. This variation occurs because some samples are inherently easier or harder for the model to predict, and therefore the model performance typically varies across random splits. There is thus a trade-off between fitting the train data and generalizing to unseen samples, which results in a negative relationship between train and test RMSE across random splits. The variation in standardized regression coefficients, *p*-values, and model predictability due to 100 random data partitions was much larger than the variation observed across the 100 subcompositions (Supplementary Figure S7). For example, the standardized regression coefficient for the genus *Negativicoccus* in the full composition was 0.0499. Across the 100 subcompositions, this value ranged from 0.0498 to 0.0499, whereas across 100 random data splits it ranged from −0.011 to 0.058. Similarly, comparable patterns were observed for *p*-values and for the training and test RMSE across all datasets. The *p*-values from the 100 data splits were generally lower than those of the reference split, which was used for feature selection, and is retained here for consistency. For the same reason, the reference split tends to show an extremely low training RMSE and a comparatively high test RMSE: being the split used for variable selection, it produces the best fit on the train set and, due to the train-test trade-off, consequently performs worse on the test set.

Figure 8 shows the subcompositional coherence under a PLS approach. The PLS models used to predict litter size, ADG or IMF were built with 3, 1, 1, 2, or 3 latent components, respectively, and included 515 ASVs, 55 microbial genera, 1,230 microbial genes, 206 transcripts, or 81 metabolites. The regression coefficients of the top five *omic* features were subcompositionally coherent in all cases. Their associated VIP values (all above 1.5) were also coherent, except for ASV2 whose VIP value was 3.21 in the full composition and ranged between 2.94 and 3.05 across subcompositions; and *Methanobrevibacter* whose VIP value was 3.01 in the full composition and ranged between 2.91 and 2.93 across subcompositions. This small deviation led to changes in the ASV rankings only, while the top-5 rankings remained consistent across the other datasets. Root mean squared errors in both the train and test sets were also coherent across subcompositions.

Figure 9a shows the subcompositional coherence obtained with the random forest approach. For the five datasets, the final random forest models after feature selection included 8, 40, 23, 43, and 27 predictors, respectively. Random forests also provide an out-of-bag (OOB) error, a RMSE computed from the bootstrap samples used in each tree, which acts as an internal form of cross-validation. Model predictability was perfectly coherent across all datasets. Variable importances for the top five predictors varied across subcompositions, but this variation could be partly attributed to the stochastic nature of tree construction in random forests. In all datasets, however, the variability in importance values due to subcompositions was smaller than the widely acknowledged variation associated with different train-test splits (Figure 9b). For example, the most influential microbial genus, *Negativicoccus*, had a variable importance of 0.0088 in the full composition, ranging from 0.006 to 0.011 across the 100 subcompositions, while the range across 100 train–test splits was wider (0.003–0.011). The feature rankings derived from these importances (Supplementary Table S1) show that the highest-ranked predictors generally remain in the top positions when compared with the full composition. Although some rank permutations occur across subcompositions, they are far more consistent than those arising from different dataset splits, which result in substantially larger ranking fluctuations (Supplementary Table S2).

Finally, the linear mixed model with a Gaussian kernel showed highly subcompositionally coherent outputs. Estimated *omic* values were highly correlated between subcomposition and full composition, the average Pearson correlations were 0.997 (ASVs), 0.999 (microbial genera) and 0.997 (microbial genes), respectively (Supplementary Figure S6). Finally, the ranges of RMSE values in the train and test sets were very narrow and almost identical in the full compositions and subcompositions. In microbial genera, RMSE train ranged [min, max] from [0.239, 0.241] in both, and RMSE test ranged from [0.289, 0.293] in subcompositions and from [0.289, 0.294] in full compositions. In microbial genes (subcompositions vs. full composition) RMSE train was [0.235, 0.245] vs. [0.234, 0.245], and RMSE test was [0.294, 0.297] vs. [0.294, 0.297]. In ASVs, RMSE train was [1.89, 2.28] vs. [1.94,2.29] and RMSE test was [2.98, 3.21] vs. [2.95, 3.14]. The *omic* and residual variance estimates had wide 95% highest posterior density intervals, preventing an accurate assessment of subcompositional coherence.

## 4 Discussion

### 4.1 Omics data are subcompositionally coherent in practice

*Omics* data are naturally compositional, and several non-compositional normalizations (TSS, TMM, RLE, CSS) are usually applied to deal with meaningless differences in sequencing library size. When a subset of features is selected and re-normalized (i.e., expressed as relative values within the new total), statistical conclusions may differ between the full composition and its subcomposition because the relative values necessarily change (Egozcue and Pawlowsky-Glahn, 2011). This phenomenon is known as subcompositional incoherence and is considered an important challenge in the analysis of relative values. The classical solution to this problem is the use of logratio transformations (Aitchison, 1986). However, the number of pairwise logratios between *omics* features becomes huge, and they also require the replacement of zeros, typically through imputation, which introduces spurious variability, especially when zeros are common, as often happens in *omics* data.

Subcompositional incoherence has been only subtly characterized in high-dimensional scenarios (Gloor, 2023), despite the fact that subcompositions are ubiquitous in the *omics* field. Here, we documented subcompositional coherence of TSS-normalized *omics* relative data across a comprehensive set of unsupervised and supervised learning analyses, performed on five already published typical datasets of different nature (16S metagenomics, whole metagenomics, transcriptomics, and metabolomics), each with distinct numbers of features, sample sizes, and compositional diversity (see Figure 1). The overall picture emerging from our analyses is that subcompositional incoherence under an abundance-weighted subcomposition scheme is greatly attenuated in TSS-normalized *omics* datasets and that subcompositions are very close to being coherent; that is, they yield almost the same results as those obtained using the full composition. We extend our subcompositional coherence evaluation to feature coherence and unsupervised approaches to the microbial genera dataset, this time using relative values after TMM, RLE and CSS transformation (see Supplementary Analyses). All three normalization methods still showed high levels of subcompositional coherence, although consistently lower than TSS. Count-based models provide an alternative framework for analyzing relative *omics* data (Anders and Huber, 2010). However, our objective was to evaluate the practical consequences of subcompositional incoherence in the transformed abundance matrices commonly used in downstream unsupervised and supervised analyses.

It remains theoretically possible that a dataset could exist in which analyses performed without logratio transformations would yield different results for the full composition and its subcompositions, for example, in an extremely small composition representing less than 10% of a highly diverse dataset. We facilitate the unsuper_subco() and super_subco() functions accessible from GitHub (see Data Availability section) so researchers can assess whether their own datasets are subcompositionally coherent. While we dismantled an important criticism of working with relative values; TSS (or TMM, RLE and CSS) normalized relative abundances need to fulfill other mathematical properties in favor of reproducibility before they can be safely recommended for the *omics* data analyses. *Omics* relative values should be scale-invariant (i.e., robust to changes in the library size) and perturbation invariant (stability towards feature-specific systematic biases), which remains to be formally assessed in realistic biological contexts that occur in the *omics* field.

Readers should not interpret our results as evidence that conclusions drawn from relative abundances are directly transferable to the corresponding absolute abundances, unless the true library size is identical across samples, which is a highly unlikely scenario. Correlations between features computed from relative abundances are known to exhibit a negative bias compared with the corresponding correlations in absolute abundances due to the constant-sum constraint (Aitchison, 1986), which in *omics* data occur successively across multiple steps. This bias exists regardless of whether the data are normalized in the full composition or in a subcomposition. For researchers interested in inferences on absolute quantities, recent work has sought to alleviate the problem of unmeasured scale by introducing probabilistic models that account for scale uncertainty, incorporating prior biological knowledge, or, ideally, directly measuring bacterial load using flow cytometry (Nixon et al., 2025).

### 4.2 The typical subcomposition case in *omics* field

When we configured how to form subcompositions, we intended to simulate the most common situations in our field. One scenario arises when, over time, new *omic* features are identified and reference databases expand, so the current datasets can be considered as a subcomposition of future more comprehensive ones, if they are reprocessed. Another scenario occurs when samples are resequenced at different depths, resulting in smaller or larger subcompositions. One more scenario arises when some features are removed from the analysis and the data are re-normalized, often due to low occurrence across samples, minimum mean-abundance thresholds, or unknown biological functions. This is common because many reads in current *omics* datasets cannot be assigned to known features or have multiple or ambiguous annotations. These filtering practices often vary across laboratories. In the three cases, we considered a conservative worst-case scenario when a subcomposition contains only one third of the features of the full composition. For example, in metagenomics, the number of identified genera ranged from 500 to 800 when sequencing depth increased from 9.67 × 10^5^ to 1.76 × 10^8^ reads (Zaheer et al., 2018); in microbial gene profiling, simulated sequencing depths of 5 million reads captured approximately 80% of the microbial genes detected at 20 million reads (Liu et al., 2022); in transcriptomics, more than 90% of annotated genes were detected in datasets with 28.7–29.6 million reads, whereas only 68% were detected with 1.6 million reads (Wang et al., 2011). In all these examples, subcompositions had sizes much larger than one third of the corresponding full composition.

Considering weighting sampling, it might be argued that we should not give more probability to the most abundant features when forming the subcompositions. However, in microbiome studies, the most abundant genera often constitute the core microbiome, a set of microorganisms that appear consistently across individuals and are necessary for normal host function (Perlman et al., 2022). Thus, it will be very unlikely to find a cow whose rumen lacks some of this microbial core (e.g., *Prevotella*). Similar core structures also exist at other *omics* levels: certain microbial genes are indispensable for microbial survival (Perlman et al., 2022); some genes defining tissue function are usually highly expressed for a given tissue (Esmaili et al., 2021); finally, every biofluid has a set of metabolites which reflects basic physiology and produce strong and consistent ion signals across samples (Ghosh et al., 2024). Because these abundant and essential components appear in any dataset, comparable or follow-up studies will inevitably include them, whereas the presence of rare features will vary depending on sequencing depth, database annotation, or experimental conditions. For the supervised analyses, subcompositions were constructed so that features identified as important in the full composition remained available. This was intended to isolate the effect of compositional re-normalization, avoiding confounding with changes in predictor availability and method-specific selection behavior. Consequently, our results assess the impact of subcomposition coherence conditional on the availability of the relevant predictors, rather than the stability of the complete analytical pipeline, where feature selection is typically performed separately in each dataset and important predictors may not always be available.

### 4.3 Relative values vs. logratios

Although log-transformations are not required to maintain statistical coherence in TMM-normalized *omics* datasets, it is a common misconception that analyses based on relative abundances and on log-transformed compositional data should yield similar results. When logratio transformations are used, the transformed variables differ from the relative ones, and have a different meaning and interpretation. This comparison has been made by different studies, on the context of different unsupervised (Bars-Cortina, 2022) or supervised analysis, including compositions as predictors (Zhang et al., 2021, 2024) or targets (Garcia et al., 2025). These reveal different results, some advocating for log-transformations (Zhang et al., 2024), others finding no differences (Bars-Cortina, 2022; Zhang et al., 2021), and others getting better results with raw relative data, identifying zero-imputation artifacts as one of the main causes of degraded performance (Garcia et al., 2025). Overall, it is difficult to generalize, and the most appropriate transformation, if needed, often needs to be determined empirically.

### 4.4 Conclusion

In this study, we have shown that the relative values of TSS-transformed *omics* datasets are almost subcompositionally coherent, as well as their results derived from a comprehensive set of unsupervised and supervised analyses. Our conclusions are subject to an abundance-weighted subcomposition scheme, which reflects the most common scenario in *omics* analyses. By demonstrating subcompositional coherence across five distinct *omics* datasets, our results suggest that concerns regarding the use of relative abundances may be less severe than often assumed, thereby challenging an important argument for the routine use of log-ratio transformations.

## Data Availability

The original contributions presented in the study are publicly available. Liver transcriptomic data from rabbits are available in the European Nucleotide Archive (ENA) under accession project PRJEB104993. The 16S metagenomic sequences from rabbit doe feces are available under accession project PRJEB83677. Rumen metagenomic sequence reads are available in the ENA under accession projects PRJEB31266, PRJEB21624, PRJEB10338, and PRJEB70912. Rabbit plasma metabolite abundances are available upon request. The functions unsupersubco() and supersubco(), used to assess subcompositional coherence, can be downloaded from https://github.com/michaelgreenacre/CODAinPractice.
