# Supplementary material for: Omics data in relative values are almost subcompositionally coherent
Source: Front Microbiol. 2026 Jul 23;17:1809364. doi: 10.3389/fmicb.2026.1809364 (PMC13441932; doi:10.3389/fmicb.2026.1809364)
Supplement: Supplementary file 1 [file Supplementary_file_1.pdf]

Supplementary Material

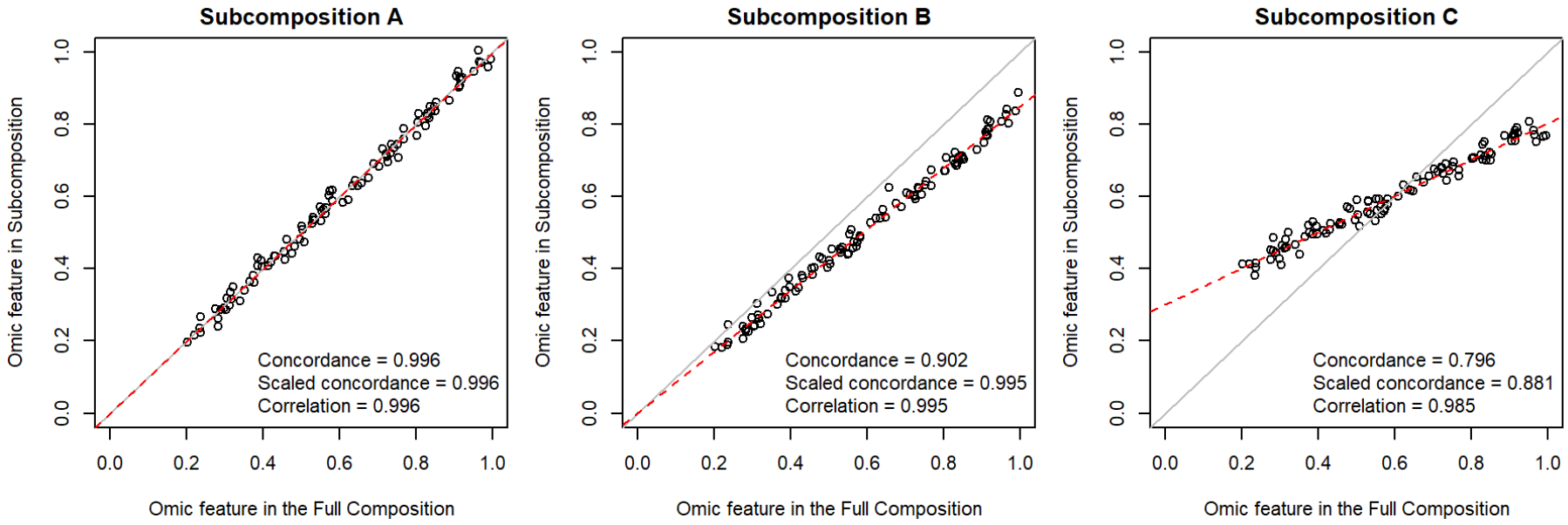

Fig. S1: Example of the three measurements of agreement, concordance (exact numerical agreement), scaled concordance (agreement allowing for scale differences), and Pearson correlation (allowing for both intercept and scale differences), between an *omic* feature in a full composition and the same feature renormalized in three different subcompositions. In Subcomposition A, all three measures are very high. In Subcomposition B, the feature shows almost perfect scaled concordance and correlation, but not concordance. In Subcomposition C, only the correlation remains very high. A 45-degree identity line is shown in all three plots for reference.

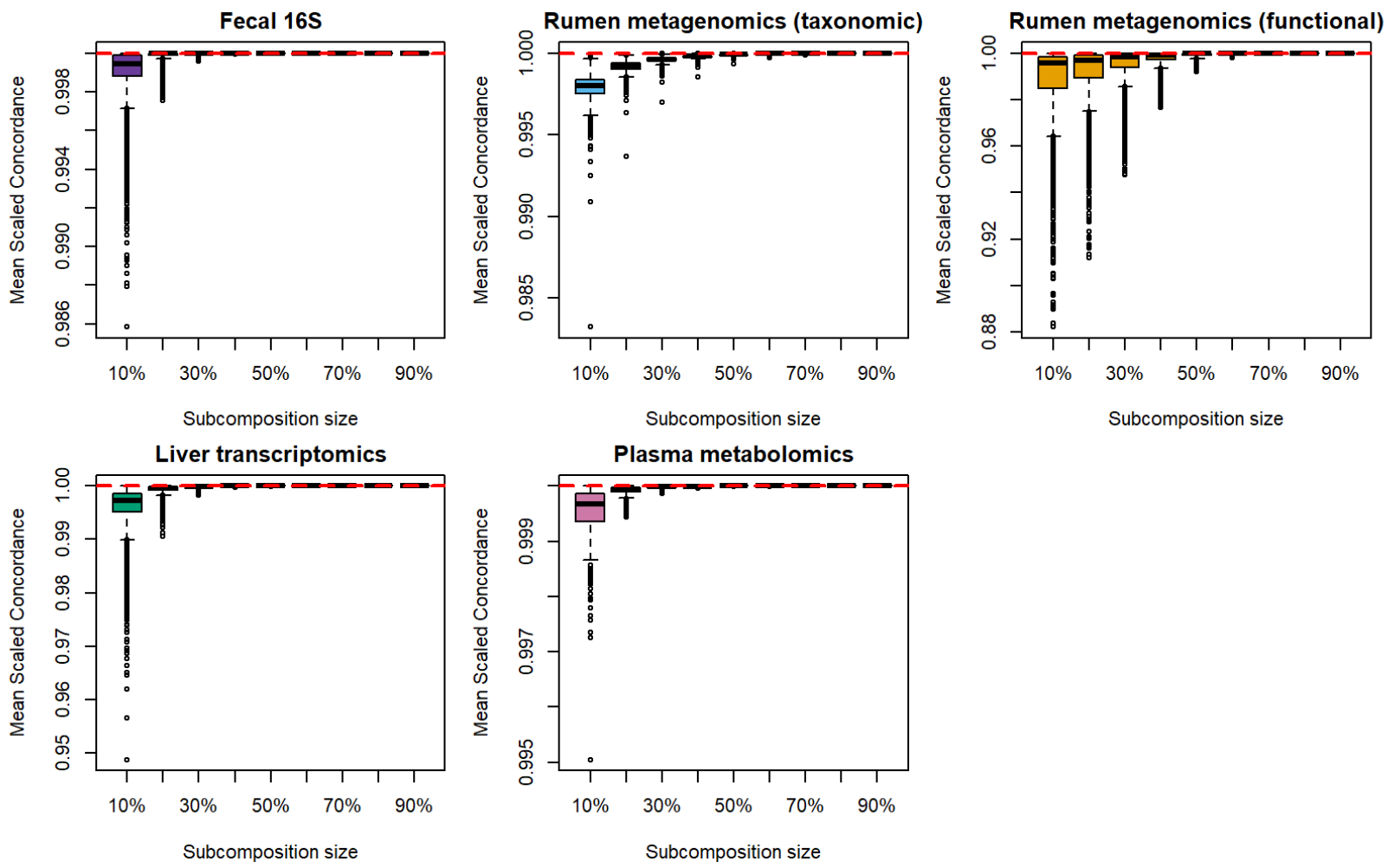

Fig. S2: **Effect of subcomposition size on feature coherence.** Scaled concordance between feature values in the full composition and in 100 randomly generated subcompositions of different sizes (10–90% of the full feature set).

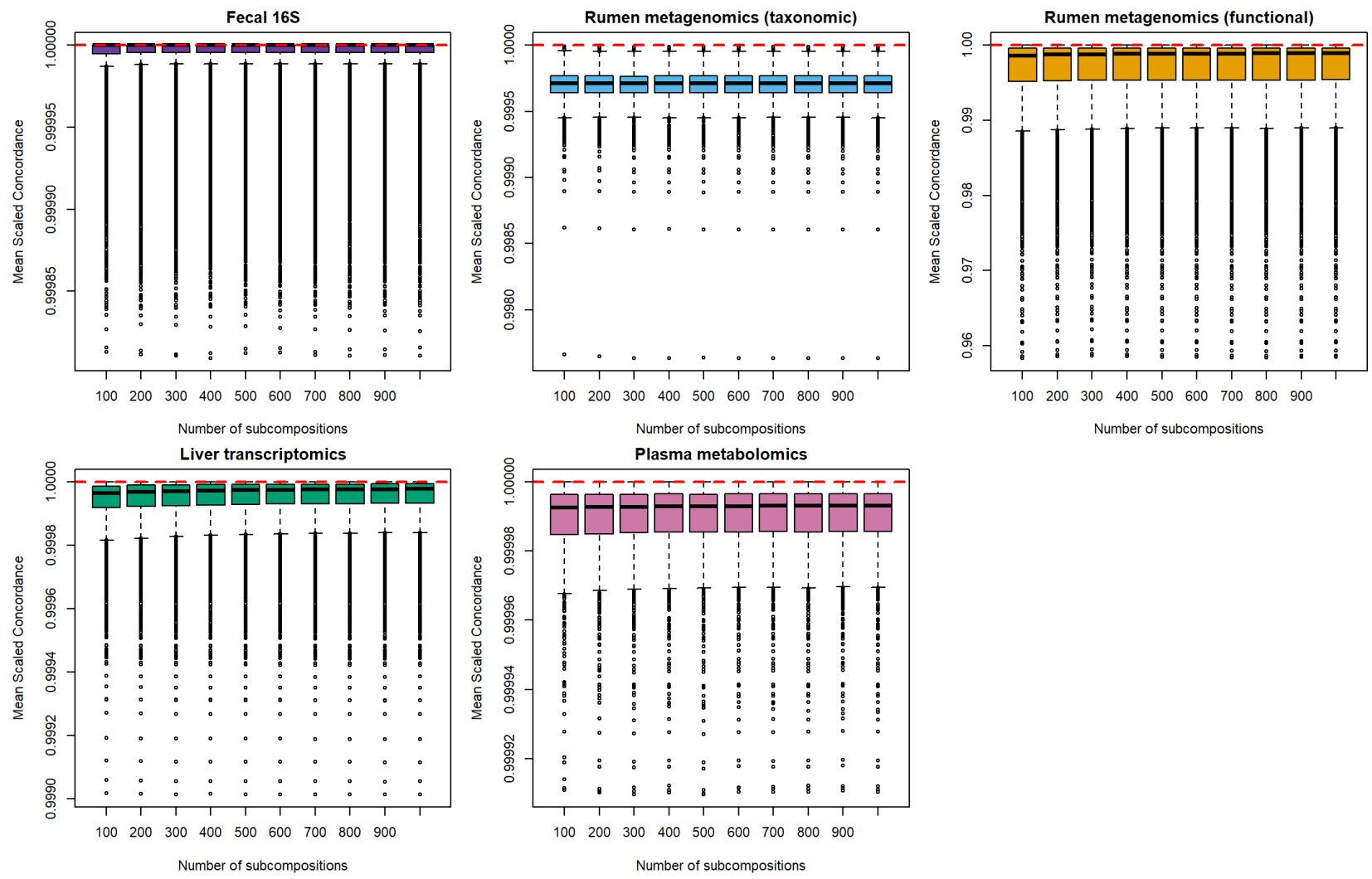

Fig. S3: **Effect of the number of subcompositions tested on average feature coherence.** Average scaled concordance between feature values in the full composition and in subcompositions, computed over 100–1000 random subcompositions (all of size one-third of the features).

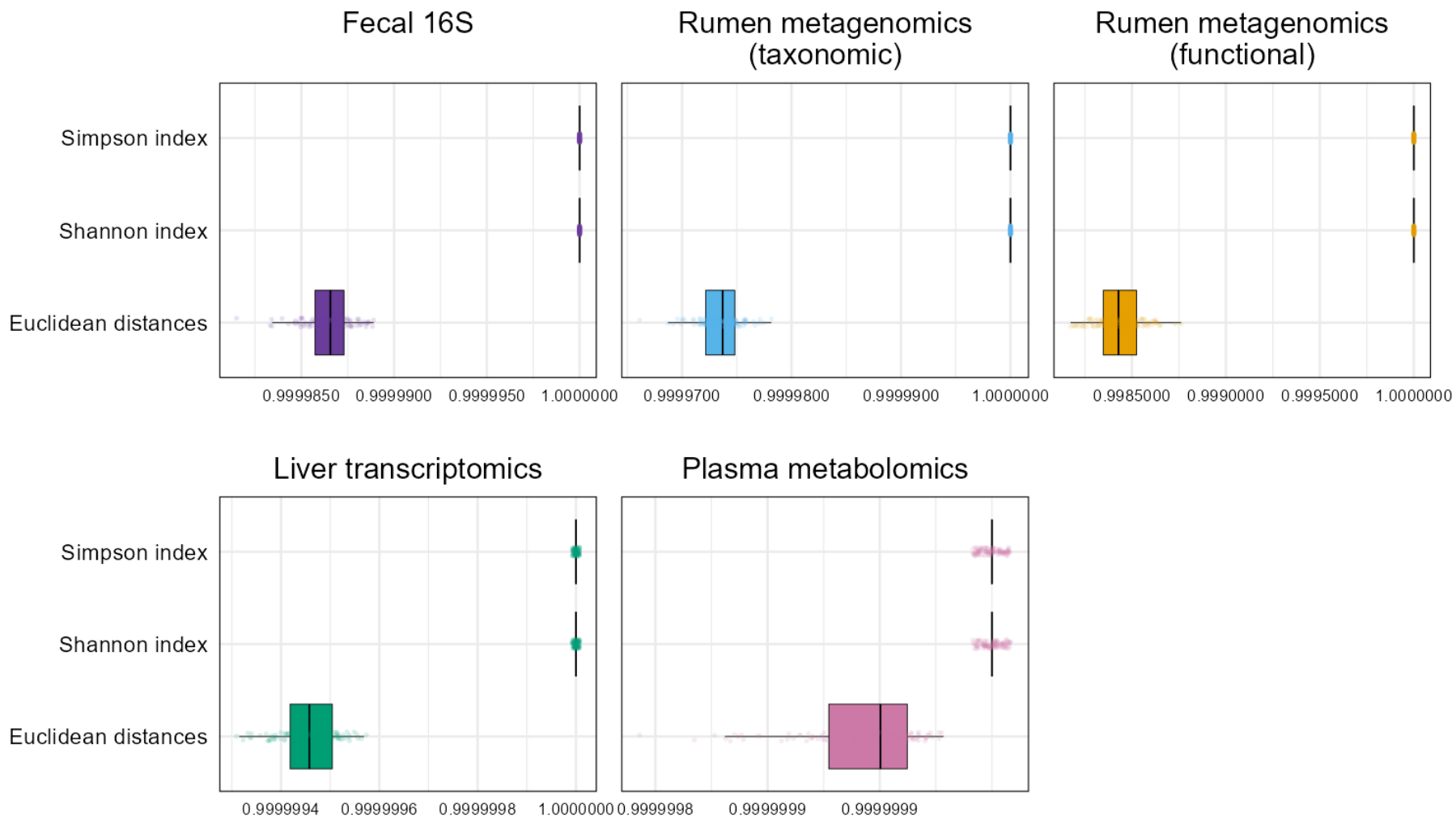

Fig. S4: **Subcompositional similarity in the diversity within and between samples.** Scaled concordances between full compositions and 100 subcompositions for Shannon and Simpson indices and Euclidean distances between samples.

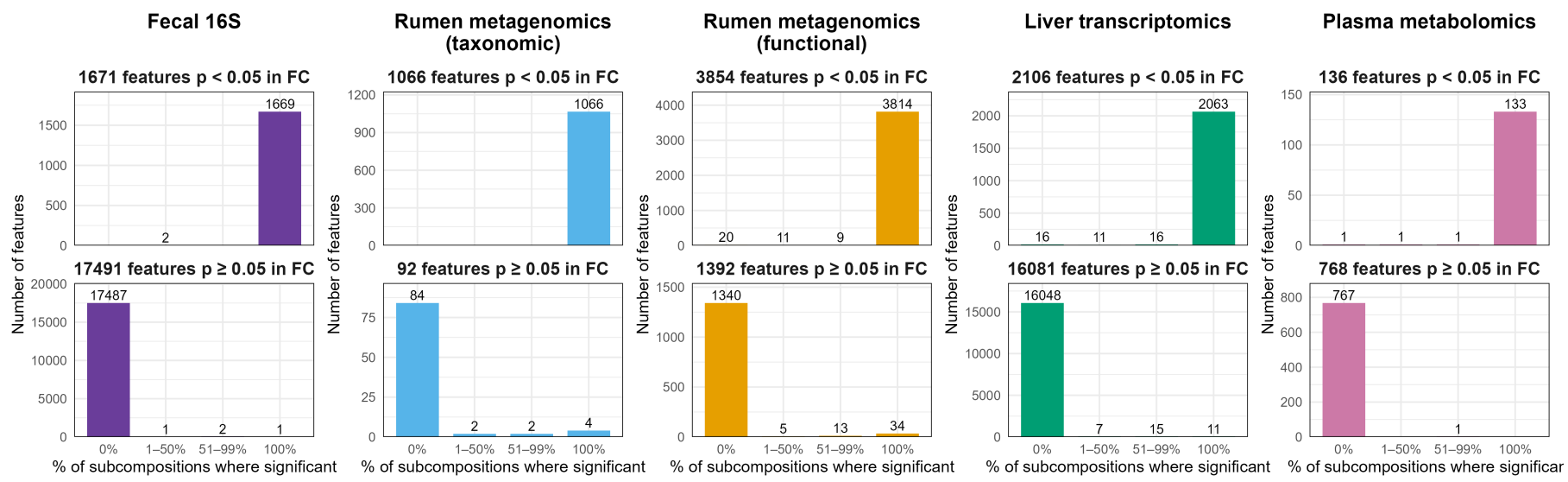

Fig. S5: **Subcompositional coherence in differential abundance analyses using t-test.** Subcompositional coherence in differential abundance analyses between seasons (ASVs), between diets (rumen microbial genera and microbial genes) or between genders (liver transcriptomics and plasma metabolomics). Features were classified as significant or non-significant using a t-test with a significance threshold of $p - value < 0.05$. The upper and lower panels show the coherence of features identified as significant or non-significant in the full composition (FC), respectively. Bars indicate the percentage of subcompositions in which these features were identified as significant in the corresponding subcompositions. Features not selected in any subcomposition are omitted.

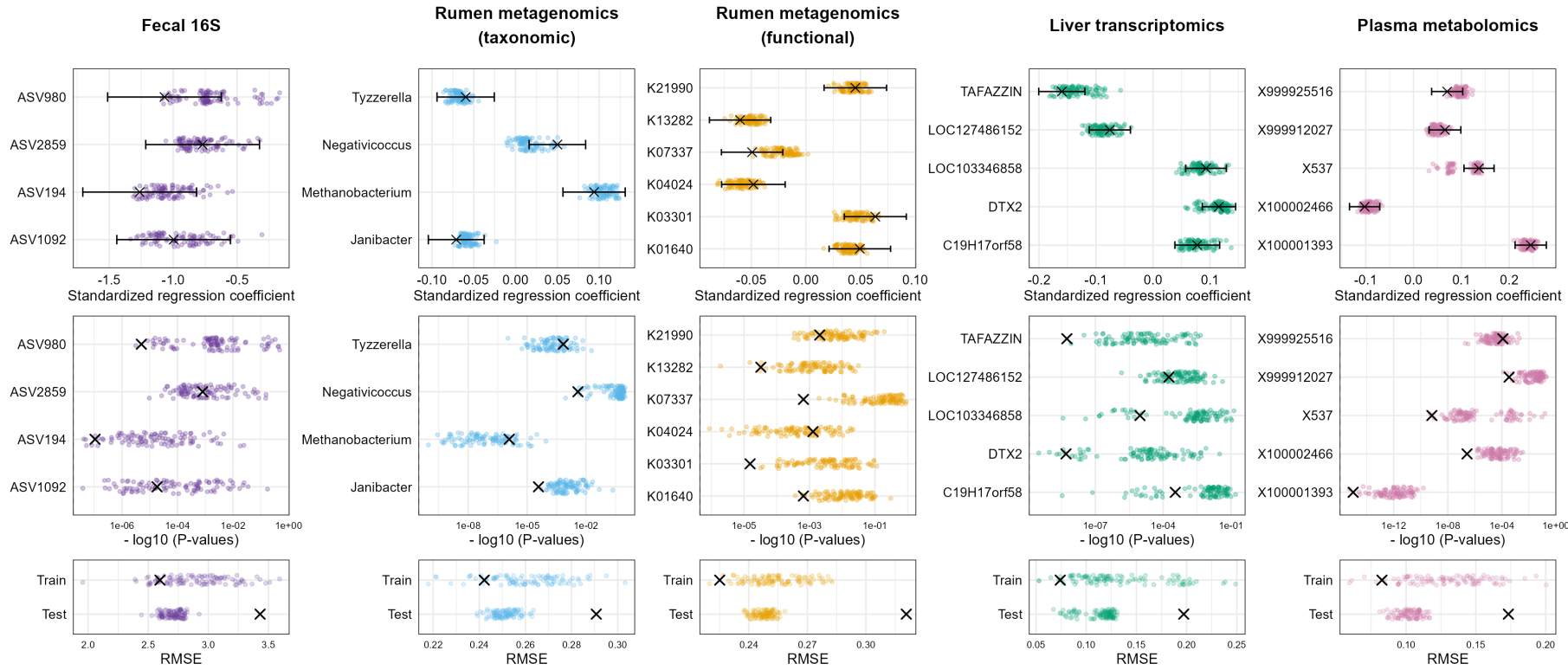

Fig. S6: **Train and test data split coherence analysis in linear regression.** Train and test data split coherence analysis in five supervised linear regression models aimed at predicting litter size using ASVs, average daily gain using ruminal metagenomics (microbial genera or microbial genes) or intramuscular fat using liver transcriptomics or plasma metabolomics. Black crosses represent the estimated effect sizes of the selected *omic* features, expressed as standardized regression coefficients, together with their 95% confidence intervals, corresponding *p*-values, and the model root mean squared error (RMSE). Coloured points show the same parameters obtained when the same model is fitted using 100 different train sets, and tested in 100 different test sets.

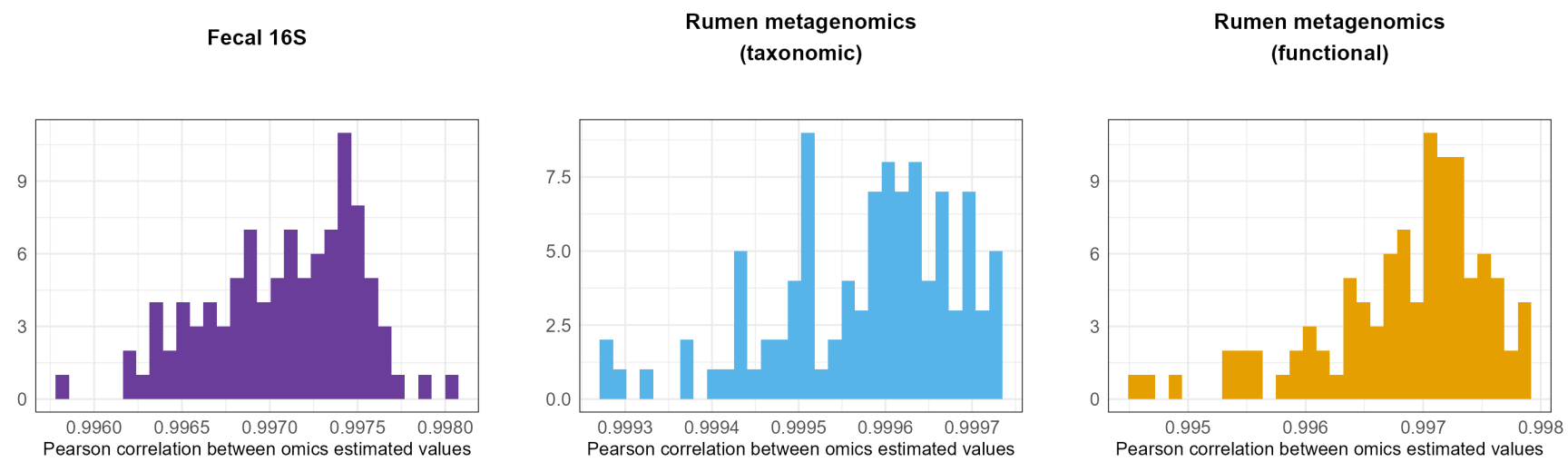

Fig. S7: Subcompositional coherence analysis in supervised linear mixed model with a Gaussian kernel for predicting average daily gain using ruminal metagenomics data (microbial genera or microbial genes). The plot shows histograms of Pearson correlations between the estimated *omic* effects obtained from each subcomposition and those from the full composition.

Table S1: **Ranks in the 100 subcompositions for the top five features in each dataset.** For each dataset, the table shows how frequently each top feature (selected by random forest importance in the full composition) appears in each rank (1–5) across 100 subcompositions.

(a) Fecal 16S

Original rank	1	2	3	4	5
1 *ASV198*	71	16	5	5	3
2 *ASV2326*	15	39	26	16	3
3 *ASV1333*	1	1	1	9	22
4 *ASV247*	6	11	6	12	20
5 *ASV2859*	1	9	26	26	21

(b) Rumen metagenomics (taxonomic)

Original rank	1	2	3	4	5
1 *Negativicoccus*	89	11	0	0	0
2 *Halococcus*	11	89	0	0	0
3 *Halopenitus*	0	0	69	28	3
4 *Thermobifida*	0	0	3	16	36
5 *Chryseobacterium*	0	0	26	49	19

(c) Rumen metagenomics (functional)

Original rank	1	2	3	4	5
1 K02948	51	36	13	0	0
2 K03313	5	29	66	0	0
3 K13727	44	35	21	0	0
4 K04024	0	0	0	4	3
5 K02887	0	0	0	1	2

(d) Liver transcriptomics

Original rank	1	2	3	4	5
1 CRYZ	41	22	15	9	2
2 PLEKHG7	25	11	14	15	13
3 TAFAZZIN	19	27	17	8	14
4 FAM91A1	2	10	10	12	11
5 LOC100349168	1	1	2	5	8

(e) Plasma metabolomics

Original rank	1	2	3	4	5
1 X100001393	100	0	0	0	0
2 X100001395	0	18	25	21	14
3 X1506	0	4	10	15	21
4 X100004523	0	33	27	18	14
5 X100001272	0	36	20	14	10

Table S2: Ranks in the 100 train-test samples split for the top five features in each dataset. For each dataset, the table shows how frequently each top feature (selected by random forest importance in the full composition) appears in each rank (1–5) across 100 data splits.

(a) Fecal 16S

Original rank	1	2	3	4	5
1 *ASV198*	4	3	7	4	13
2 *ASV2326*	16	19	11	10	13
3 *ASV1333*	0	4	10	8	8
4 *ASV247*	13	29	21	14	9
5 *ASV2859*	11	10	14	18	14

(b) Rumen metagenomics (taxonomic)

Original rank	1	2	3	4	5
1 *Negativicoccus*	50	32	9	5	0
2 *Halococcus*	35	47	13	4	0
3 *Halopenitus*	0	1	4	4	5
4 *Thermobifida*	0	0	1	3	3
5 *Chryseobacterium*	0	1	4	7	12

(c) Rumen metagenomics (functional)

Original rank	1	2	3	4	5
1 K02948	18	26	17	13	4
2 K03313	0	0	2	0	0
3 K13727	8	5	5	6	10
4 K04024	13	9	7	8	6
5 K02887	1	0	0	2	2

(d) Liver transcriptomics

Original rank	1	2	3	4	5
1 CRYZ	12	5	5	2	5
2 PLEKHG7	3	3	2	4	1
3 TAFAZZIN	0	1	2	3	1
4 FAM91A1	1	0	1	1	3
5 LOC100349168	20	14	5	10	8

(e) Plasma metabolomics

Original rank	1	2	3	4	5
1 X100001393	63	19	5	5	3
2 X100001395	18	29	32	12	3
3 X1506	6	23	17	18	14
4 X100004523	0	1	0	0	1
5 X100001272	3	2	7	12	5

Supplementary Analysis

In this paper we have dealt exclusively with the coherence of compositional data subject to normalization by total counts, called total sum scaling (TSS). Any other normalization can be evaluated for coherence in the same way, using the same measures that we have proposed.

In the following we present some results for compositional relative values after normalizations RLE (relative log-expression), TMM (trimmed mean of M-values) and CSS (cumulative sum scaling), applied to the first of our data sets, the rumen metagenomics (taxonomic) data. Transformations TMM and RLE were computed with edge R package (Robinson et al, 2010) and CSS with metagenomeseq R package (Paulson et al, 2013). The procedure is the same: first, perform the normalization on the complete data set and retain some results; second, select 100 random subcompositions of one-third of the features (genera in this case), renormalize this reduced data set and retain the same results; third, measure the agreement between the latter results for the subset and those of the corresponding results from the full set. As an example, we measured the agreement using the concordance between the correlations, the Procrustes correlation between the feature geometries, and the scaled concordance of the features. The results are summarized by the median and ranges of these three measures for the two normalizations (Table **??**). The results for the TSS normalization used in this paper are also given as a comparison.

Table S3: Subcompositional coherence for microbial genera relative values under different normalizations. Values are median [range].

Microbial Genera	TSS	TMM	RLE	CSS
Pearson correlation concordance	0.9994 [0.9993, 0.9995]	0.9864 [0.9607, 0.9946]	0.9810 [0.9583, 0.9939]	0.9764 [0.8401, 0.9935]
Procrustes	0.9988 [0.9986, 0.9995]	0.9997 [0.9993, 0.9998]	0.9997 [0.9995, 0.9999]	0.9996 [0.9953, 0.9998]
Feature-scaled concordance	0.9997 [0.9977, 0.9999]	0.9816 [0.8891, 0.9998]	0.9912 [0.8967, 0.9998]	0.9844 [0.8926, 0.9998]

Among the normalization methods evaluated, TSS, which is treated in this paper, showed the highest subcompositional coherence across all metrics. TMM, RLE and CSS also showed generally high coherence, although consistently lower than TSS. Mean feature-scaled concordance remained high under these methods with medians ≥ 0.98, but minimum values dropped to around 0.89 for some features. Similarly, the mean Pearson correlation concordance between persons remained high, around 0.98, with minimum values being higher under TMM and RLE, around 0.96, than under CSS, around 0.84.
